# Improving the quality of prison research: A qualitative study of ex‐offender service user involvement in prison suicide prevention research

**DOI:** 10.1111/hex.12590

**Published:** 2017-06-22

**Authors:** Yvonne F. Awenat, Currie Moore, Patricia A. Gooding, Fiona Ulph, Aisha Mirza, Daniel Pratt

**Affiliations:** ^1^ Division of Psychology and Mental Health School of Health Sciences University of Manchester Manchester UK; ^2^ Manchester Academic Health Science Centre, MAHSC Manchester UK; ^3^ Institute of Brain, Behaviour and Mental Health University of Manchester Manchester UK; ^4^ Centre for New Treatments and Understanding in Mental health CENTRUM University of Manchester Manchester UK; ^5^ Manchester Mental Health and Social Care NHS Trust Manchester UK

**Keywords:** ex‐offenders, prisoners, qualitative, suicide, user‐involvement

## Abstract

**Background:**

Suicide is the leading cause of avoidable death in prisons worldwide and suicide prevention is an international priority. Consequently, there is an urgent need to develop evidence‐based treatments. We conducted a randomized controlled trial of a novel suicide prevention psychological therapy for male prisoners. To promote ecological validity by addressing the “real‐world” situation of suicidal prisoners, we involved a consultant group of ex‐offenders with past experience of being suicidal during imprisonment. Service user involvement in prison research is challenging and underdeveloped.

**Objective:**

We aimed to investigate the ex‐offender service user consultants’ experiences of being involved in the research.

**Design:**

Individual qualitative interviews were conducted and analysed using an Interpretative Phenomenology Analysis (IPA) framework.

**Setting/participants:**

The study was conducted at a university in North England, UK, comprising four ex‐offenders with experience of being suicidal during past imprisonments.

**Results:**

Two superordinate themes were identified: “Working Together” depicted participants’ perceptions of the pivotal role of good relationships with researchers, and “Journey of Change” outlined how participants’ involvement in the research impacted on their personal lives.

**Discussion:**

Little is known about how to successfully involve ex‐offender service users in research. Our results indicate the conditions necessary for successfully engaging ex‐offender service users in research and have important implications for improving the quality of prison research.

**Conclusions:**

Involving forensic service users in research is feasible and should be encouraged, as despite certain challenges, it is highly rewarding both for the research and the ex‐offender service users.

## 1 INTRODUCTION

Suicide is a global health problem in custodial and correctional institutions being cited as the single most common cause of death for incarcerated people.^1^ A study examining prisoner suicides in twelve countries found rates in Western Europe higher that in Australia, Canada and New Zealand.^2^ In the United States of America, suicide is the leading cause of death in local jails and state prisons, representing 34% of all inmate deaths.^3^ In England and Wales, suicide is the leading cause of preventable death in prisons with prisoners being eight times more likely to die by suicide than the general populations.^2^, ^4^ During 2015 to 2016, self‐inflicted deaths by prisoners in England and Wales increased by 28% to an unprecedented high rate of 1.2 per 1000 prisoners.^5^ Consequently, suicide prevention in prisons has been hailed as an international health priority by the World Health Organization,^1^ and similarly is a priority for the UK Ministry of Justice^6^ and the National Health Service^7^, ^8^ who are responsible for UK prison health care.

The Prevention of Suicide in Prisons (PROSPeR) study was a three‐year clinical trial investigating the feasibility and acceptability of a novel Cognitive Behavioural Suicide Prevention therapy^9^ specifically targeting suicidal thoughts and behaviour in prisoners, the findings of which are reported elsewhere.^10^ An important component of the study was the involvement of a Service User Reference Group (SURG) comprising ex‐offenders with “lived experience” of suicidal thoughts or behaviour during imprisonment. To our knowledge, this is the first ex‐offender SURG to be substantively involved in prison suicide prevention research in the United Kingdom.

Few people, other than those who either work in, or have been incarcerated in, a prison have any authoritative “lived experience” of the realities of prison life.^11^ Service user involvement in research is well established within the UK Department of Health policy,^12^, ^13^, ^14^ being recognized as a hallmark of good research practice in many contemporary fields of study.^15^, ^16^ However, service user involvement in prison research remains underdeveloped^17^, ^18^ with little published research examining how to engage and retain individuals with experience of imprisonment in the research process.^19^, ^20^, ^21^ Furthermore, from the small corpus of existing publications, some studies purporting to describe prisoner or ex‐offender service user *involvement* have employed research methodologies where service users were research *participants* rather than collaborators.^22^ This deviates somewhat from the definition of involvement stated by INVOLVE^16^ (p7) as “research being carried out ‘with’ or ‘by’ non‐professionals rather than ‘to’, ‘about’ or ‘for’ them.”

Conducting research in prisons is challenging due to the complexity of contextual variables unique to prison settings.^23^, ^24^, ^25^, ^26^ Barriers to successful implementation of prison research include the necessity to work within stringent security regulations and the prevailing organizational culture and attitudes of prisoners and staff.^27^ Given the uniqueness of the prison setting and the lack of contextual knowledge accessible to mainstream researchers, it is especially important to involve individuals with “lived experience” of imprisonment as service user advisors or consultants to bring a “real‐world” perspective to the design and implementation of prison research. The purpose of this study was to investigate and understand SURG member's experiences of being involved in the PROSPeR study.

### 1.1 The service user reference group

Recruitment of ex‐offenders with experience of being suicidal during imprisonment was achieved by phone or email approaches to organizations likely to be in contact with ex‐offenders (e.g probation services, prison charities, user groups). Those willing to help were then asked to circulate a poster inviting interested ex‐offenders to a meeting at the university where information was provided about the study and SURG. Seven people attended the meeting following which they were invited to meet research staff individually to ascertain their possession of the required “lived experience” and ability to attend meetings regularly. This resulted in five people wishing to join the SURG; however, one member dropped out after two years for reasons unknown.

Throughout the three‐year study, four SURG members attended monthly research meetings at the university to guide the research team throughout all aspects of the research process. The SURG was founded on the collaborative model of involvement advocated by INVOLVE^16^ whereby contributions of the researchers and SURG members held equal value despite emanating from different perspectives. Further details concerning the recruitment strategies and operational structures and processes we developed are described elsewhere.^28^

## 2 METHODS

### 2.1 Design

In‐depth individual qualitative interviews, following Interpretative Phenomenological Analysis (IPA) methodology,^29^ were conducted. We aspired to elicit the particular subjective experiences of each SURG member in accordance with IPA's ideographic focus.^30^

### 2.2 Recruitment

Ethical approval was obtained from the university's Research Ethics Committee (reference number 11473). Recruitment and data collection were conducted by CM who was independent from the PROSPeR research team. Information about the study was offered by CM at a SURG meeting.

### 2.3 Eligibility

Inclusion criteria specified current membership of the PROSPeR SURG and ability to provide informed consent. There were no specified exclusion criteria.

### 2.4 Participants

To preserve the anonymity of this unique sample, we have limited the provision of demographic data, and participants are identified by a pseudonym. All four SURG members gave written consent to participate. The sample had an equal gender split, mixed ethnicity and age range of 40‐60 years old. Past imprisonment ranged from 1 to 28 episodes equating to almost 50 years collectively.

### 2.5 Procedure

Interviews were carried out at the university at times convenient to participants. A semi‐structured topic guide (available upon request) posed questions to initiate conversations about participants’ experiences as SURG members. Questions investigated participants’ motivations and expectations of joining the SURG, views about how they were involved and how involvement had impacted on them personally. Questions also probed participants’ views about relationships with fellow SURG members and the research team, along with any challenges or disappointments encountered. Interviews lasted approximately one hour (range 50‐66 minutes) following which they were transcribed verbatim by the interviewer who removed all identifying information.

### 2.6 Analysis

As advocated for IPA, each transcript was analysed individually.^31^ Several readings of each transcript preceded “initial noting” of descriptive comments which were then coded to form “emergent themes.” Clusters of related themes were then abstracted into superordinate and subordinate themes. This was repeated for each transcript prior to searching for similarities, idiosyncrasies and patterns across the entire data corpus. The first and second authors each carried out these procedures independently before meeting together to review interpretations. Final interpretations were agreed with all research team members.

## 3 RESULTS

**Figure 1:**
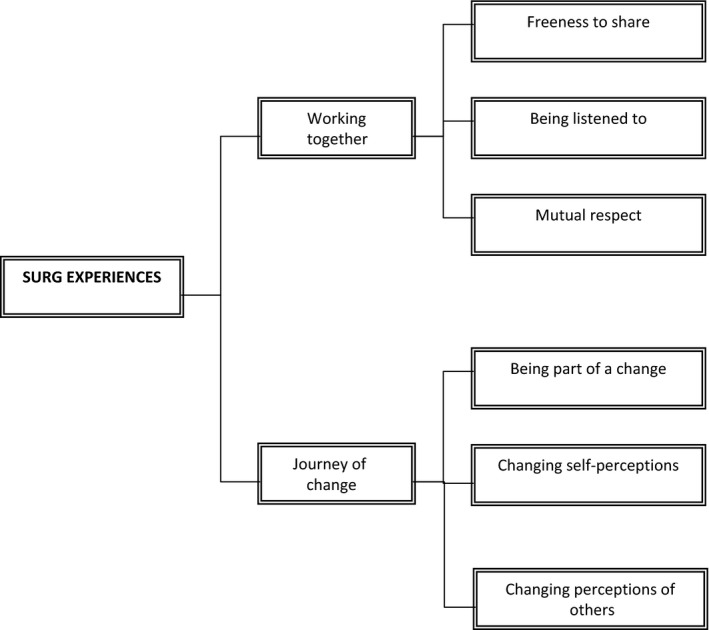
Overview of themes

There were two superordinate themes: (i) “*Working Together”* captured participants’ perceptions of factors attributable to their successful working relationships with researchers; (ii) “*Journey of Change”* depicted SURG members’ personal journeys as they embraced both the challenges and opportunities encountered. Each superordinate theme comprised three subordinate themes (See Figure 1).

### 3.1 Superordinate theme 1: working together

Three subordinate themes described participants’ perceptions of the conditions during meetings that enabled strong working relationships to flourish: (i) “Freeness to share,” (ii) “Being listened to” and (iii) “Mutual respect.”

#### 3.1.1 Freeness to share

“*Freeness to share”* was perceived critical to successful working relationships with researchers being crucial for open discussion of past encounters with suicide during imprisonment. The flexible informal atmosphere during meetings facilitated a congenial environment:

It's more relaxed. I mean informal. I think when it's too formal it stifles things cuz you get people who possibly have things to contribute but they sometimes don't because the formality of it. (Sam)

Participants appreciated researchers’ tolerance of “street language” during meetings which helped to achieve free and open communication:

…there are a few swear words that come out when we get enthusiastic or if we get emotionally going… I like how we are in PROSPeR, we all just come out with anything. (Liz)

“Freeness to share” captured participant's views of the important conditions within meetings that enabled unfettered communication of the realities of life for suicidal prisoners.

#### 3.1.2 Being listened to

Participants’ past experiences within other groups was not always positive. This influenced their expectations and concerns about involvement with PROSPeR. Being able to talk freely and feel “*listened to”* was a key concern. Sam reflected back to his thoughts during initial SURG meetings:

I had my own ideas, which thankfully have been disproved. Sometimes you're thinking to yourself that people [*service users]* are just there in a tokenistic sort of way, where with this, I've not felt that… They listen to what you say. (Sam)

Liz described how being listened to also translated into perceptions of “*being heard”*:

You know from the day that we had the interviews to now they've always listened, they always take their time – they don't butt in; they always let you finish… They don't try to put words into your mouth. It's what you say is what they hear. (Liz)

Equally important with being heard was witnessing their advice being *acted* upon:

You hear a lot of professionals that are talk, talk, talk … this is different. You talk and they do. They make things happen. (Kim)

Participants expressed delight when seeing evidence of the impact of their advice, for example concerning prison language:

There was a point early in the research and *[researcher]* said, “Oh well, I went back to his pad” and I thought as soon as she said ‘pad’ I thought, “Wow, she's getting it! That's prison jargon. She's getting it – it's coming naturally to her now.” (Ted)

Expectations of involvement with the study are likely to have been influenced by participants’ past experiences of imprisonment, where some felt that their views were neither sought nor respected. Being “listened to” was perceived as a welcome, albeit unexpected, surprise.

#### 3.1.3 Mutual respect

Establishment of *Mutual Respect* appeared fundamental to formation and sustainment of successful working relationships, especially as participants had past experiences where the stigma of their ex‐prisoner status had impacted negatively on interactions with authority figures. Participants perceived that their expertise was genuinely respected and valued by the research team, this being supported by a sense of “mutuality.”

And without each other it won't work… They [*researchers]* are talking to the people that they are researching, which I find is the most important thing on this SURG – the input of both… without each other you change nothing. Together we'll change the world. (Ted)

Participants felt respected when consulted for their specialist knowledge.

… they always come to us and asked our advice before they've gone ahead with anything. So, it made you feel so inclusive instead of, “Right, we propose this. What do you think?” And then off they go, basically. It was, you were involved, your involvement, they let you know basically it's crucial. (Kim)

Participants valued being treated non‐judgementally: “…being seen as just a person, as someone that could give something and not be judged because you've been in jail.” (Liz)

Being respected as colleagues laid the foundation for the success of the group. One way that SURG member's reciprocated respect was evident in their commitment to attending monthly meetings:

I've never missed it. It's [*SURG meetings*] something close to my heart… So … I won't cross it with anything else… I respect them and they respect us. And we've got respect for each other. (Ted)

Participants alluded to prior experiences of stigma where other peoples’ knowledge of their past criminality would result in rejection. By contrast, within the SURG, they perceived feeling respected as valued colleagues, reciprocating respect through their commitment and passion to support the research.

### 3.2 Superordinate theme 2: journey of change

*Journey of Change* comprised three subordinate themes: (i) Being a part of change; (ii) Changing self‐perceptions; and (iii) Changing perceptions of others. Participants related examples of how their involvement with the project positively impacted on their personal lives through discovery of a new identities built on expertise gained from past imprisonments.

#### 3.2.1 Being a part of change

Kim described her perception that involvement with the SURG had opened up the opportunity to pursue a different life direction whilst also alluding to a desire to make amends to society.

For all of us we've been on a journey. Our lives are completely different before, and by coming to be part of this group it's been – I'd say the journey has been totally different because we've all got that experience now that we've changed our life and we want to put something back. (Kim)

All participants had witnessed prison suicide. Consequently, their passion to help develop new treatments for suicidal prisoners defined their motivation to be involved with PROSPeR.

…it really interested me *[PROSPeR]* because while I was in prison I did have quite a few guys that I knew commit suicide. And this [*PROSPeR*] came up and it struck a chord with me, it's something that I feel really passionate about. (Sam)

Such narratives were spoken in a quiet solemn tone portraying sadness yet infused with a strong determination to drive changes and help others.

For some, involvement in the research also represented a landmark of personal change and reform. Ted described this in terms of climbing a ladder from the “bottom to the top”:

There's me looking at the street at the top of the stairs *[in the University]* going, “From a villain, to prisons, to University!” And I'm looking around going, “Whoa!” This is a big shock, you know. (Ted)

The tone and energy of Ted's language indicated his pleasure but equally his incredulity that his past criminal career had paradoxically led him into a different career within academia.

#### 3.2.2 Changing self‐perceptions

Self‐perceptions gradually changed as participants became aware that they possessed a valued specialist body of knowledge and expertise gained from their past experiences of imprisonment from which they now had something to offer society:

It *[SURG]* gave me a boost and it made me stop living thinking “You're stupid, you're daft; you're not going to go anywhere.” Start believing that, you know other people believe in ya so if other people believe in you, you start believing in yourself… And because of *[researcher]* and all and the other members of SURG, because we all believe in each other, I started believing in myself – that I deserve to be here, and I do believe that I deserve to be here.(Liz)

Participants reflected how involvement with the study generated recognition that from unwanted negative experiences of imprisonment emerged a knowledge and skill set of value to others. Of particular importance was being recognized and valued by external agents (i.e university researchers), which appeared to strengthen and validate participants’ new self‐perceptions:

And for me to have that self‐realization as well, you know “Kim, you've got skills; you've got skills.” And by somebody professional seeing that in me, that and it builds my confidence so much as well. (Kim)

Kim elaborated on how her new self‐identity as a SURG member had replaced previous identity and affiliations with “ex‐prisoner” recidivists.

It's not every ex‐offender who has that same output or outlook… because I know other ex‐offenders and they're still doing whatever it is that they do. … whereas for us, I think that with each and every one of us who is part of this group, we have all changed our lives in a positive way by being part of this group. (Kim)

For Liz, involvement in PROSPeR triggered a process of challenging negative self‐perception that her past would inevitably define her future. This led to a more optimistic future based on perceptions of herself as someone of value who can make a meaningful contribution to society:

Since joining PROSPeR now I don't live in it (*the past)*, I look forward to what I've got to give and my input and my future and where it's going, and that's what PROSPeR has given me…. It's given me a new lease on my life, it's given me structure, it's given me new friends and new family, it's given me something to look forward to. (Liz)

Kim also considered how, paradoxically her past unhappy experiences of imprisonment were now more favourably reconceptualized in recognition of how they led to her involvement in PROSPeR:

… Wow! You know it's been positive because they *[researchers]* have helped us draw out the positive sides of being in prison even though it's one of the worst things that can happen to you. (Kim)

Three of the four participants attributed their involvement in PROSPeR to having positively influenced their self‐concept enabling radical life‐transforming changes. However, this was not so for Sam, who had always maintained his innocence and wrongful imprisonment.

#### 3.2.3 Changing perceptions of others

*Changing perceptions of others* emerged in response to recognition that people who had previously known them as criminals appeared now to have changed how they perceived and responded to them. For example, Ted stated that whilst his family at first disbelieved he had genuinely changed, this altered when he showed them his University honorary staff identity card:

That honorary badge was like “Woo!” – the best thing in the world!…. To have a badge with my name and the University. Cuz everybody thought that when I recovered first *(from addictions)*, “Oh he's scamming something…. he's just scamming for loads of money.” Now they're only just coming around to believe it that I'm in it for the reason I've been saying…. They know that I'm a born again ‐ not Christian, born again villain! (Ted)

The “loudness” of Ted's often humorous words portrayed his pride and pleasure of holding a University honorary staff card which he perceived as evidence of his reformed character. The external recognition of being given honorary staff status also had a positive impact on other participants:

It *[university]* was scary because I don't have any qualifications….to be in university and to be with other lecturers… and to be treated like – we have honour passes [*identity card*]… it makes you feel special like you… are appreciated by the university. (Liz)

Honorary contract – wow! You know I feel good saying that I'm an honorary member of staff at the *[name of university]* because people do look on you in a different light… So yeah, it's amazing. And it makes me feel so good about myself. And I'm glad – I'm very proud to have that you know. To know and yeah I'm an honorary staff member. (Kim)

Sam however, who already held a university degree, was somewhat dismissive of the value of the honorary contract implying that it held little significance for him:

I mean some people wear their badge with pride… They think it's of more value than it actually is. You can go and use the library and whatever. But that's all it is like. (Sam)

However, although Sam did not allude to any changes in himself from his involvement in the SURG, he had observed changes in other members:

They are different people to the people that I first met a couple of years back. You know they really have changed. (Sam)

Perceptions that others would hold negative beliefs about them, especially officials from police and prison communities, presented a particular challenge to participants when encountering such people in their roles of service user consultants. Liz explained how this compounded her shyness when giving her first PROSPeR seminar presentation:

I was that nervous I could do nothing but laugh. When it came to my part, I could say it but I could hear it in my voice how nervous I was. Because I couldn't look at people, my head was down and just like, we have police officers here, prison officers from *[the prison she had previously been in]* that we invited, and it was like oh my Lord I felt so embarrassed. … (Liz)

Yet, paradoxically, when Liz received praise rather than disapproval from prison staff audiences, it stimulated improved self‐confidence to undertake training and get involved in further presentations:

…but then I did a small course on … confidence building. And the next time we came, I went to speak, I couldn't shut up. (Liz)

## 4 DISCUSSION

This study investigated the experiences of a small group of community‐dwelling ex‐offenders in their role as *word deleted* **“**forensic” service user consultants for the PROSPeR study. Our findings have relevance for clinicians, prison and forensic researchers and for criminal justice professionals for two reasons. First, they provide information of successful approaches for engaging and working with potentially disenfranchised ex‐offender groups per se. Second, they indicate markers of effective ways of utilizing ex‐offender service users’ personal experiences to enhance the quality and rigour of research.

The interview schedule was designed to offer participants an opportunity to discuss their experiences of involvement with the study. During interviews, participants chose to focus on talking about experiences of engaging with the research team and how this positively impacted on their future life direction, even influencing crime desistance behaviour.

Participants perceived their positive relationship with researchers as paramount to their sustained engagement with the study, with this becoming the linchpin by which they evaluated the success of their involvement. Involvement precipitated a personal journey of change culminating in reappraisal of self‐perceptions and formation of new self‐identities. During interviews, it became evident of how strongly the stigma of past imprisonments had infiltrated participants’ self‐identity and expectations for themselves and others. Consequently, participants’ focus on talking about their changed self‐identity largely concerned movement from offender to non‐offender status.

Participants described how involvement in the study precipitated an internal process of reflection whereby they challenged and rejected their criminal identity to assume a new “changed person” identity. This was an unexpected outcome of their involvement with the study both for the researchers and the ex‐offenders. Goffman's^32^ seminal works on stigma postulated how labelling of deviants leads to “spoiled identity.” Subsequently, Lemert^33^ described how societal “labelling” of criminals often leads to the labelled individual's acceptance and internalization of self‐stigma based upon the perceived views of others. Conversely, “delabelling”^34^ as a precursor of “changed person” status is professed to carry greater impact when validated by “certification” from respected officials.^35^ This may explain the high acclaim with which participants held their university honorary staff contracts.

Training is recommended to empower service users to make a meaningful contribution to research^36^, ^37^. Therefore, SURG members were given honorary contracts to enable access to university educational resources. However beyond providing training, the honorary staff identity card was valued by SURG members as a “badge of honour” marking their acceptance and status by a respected official institution. It was also used as “proof” to convince sceptical others that old criminal lifestyles were redundant.

People with criminal backgrounds are traditionally considered “hard to engage” in formal processes,^38^ yet in our study, high levels of sustained engagement and unrelenting enthusiasm for working with a university‐based research team were evident. Ex‐offenders are a recognized, marginalized and stigmatized societal group^39^ and as our participants had extensive criminal backgrounds (collectively approximately 50 years of imprisonments), they are likely to have experienced stigma and the “invisible punishments” described by Travis^40^ of societal rejection persisting beyond prison sentence completion. Feeling accepted and valued by researchers, without judgement of their past misdemeanours, appeared highly influential to participants’ sustained engagement in the study. Indeed, engagement in new prosocial roles marked by strong affiliation with an institution has been found to be influential in maintenance of desistance.^41^ Being involved with PROSPeR appeared to provide a dynamic change force epitomizing a “turning point”^34^ influential to supporting participants’ desistance.

Although the value of developing a positive environment to promote service user involvement has been described for forensic^42^ and general health populations,^37^, ^43^ our experiences suggest that this may assume greater importance for ex‐offender groups.

Motivation to become a service user consultant in research is often driven by a passion to “make a difference”^44^, ^45^ or to “give something back”,^43^ and these ideals were clearly evident for SURG members. Participants’ experiences mirrored a process of post‐traumatic growth whereby positive personal “growth” results from traumatic events.^46^ Participants described how they reconceptualized past negative experiences of imprisonment in terms “making good out of bad” recognizing that it had enabled them to help others and to also help themselves. Positive psychology approaches that foster opportunities for post‐traumatic growth within offender rehabilitation programmes have been described.^47^ Interestingly, whilst the narratives from Ted, Kim and Liz suggest experiences of post‐traumatic growth, this was not the case for Sam, who as a past university graduate possessed higher levels of self‐confidence prior to joining the SURG. Other SURG members viewed Sam as an esteemed member of the group although he did appear somewhat set apart from the others. For example whilst other members valued their university honorary contract, Sam was relatively disdainful of its impact for him. Qualitative research aims to elicit rich data across a range of perspectives as opposed to seeking congruence of views and, as such, Sam's comments represent a negative case example.^48^

The personal impact of participants’ involvement in research accords with that of non‐criminal service user consultants. Service user consultants for cancer research also described personal impacts of changed self‐perceptions, new identities and self‐reconfiguration enabled by learning new skills and developing alternative careers in cancer research.^49^

Delivering complex interventions such as psychosocial therapies to address suicidal thoughts and behaviours involves a range of health professionals, including psychiatrists, clinical psychologists and mental health nurses and requires the greatest rigour in terms of an evidence base. RCTs are recognized to be the “gold standard” evidence base for healthcare interventions.^50^ A key finding of our study is that RCTs must be carried out collaboratively, and in tandem with service users who have relevant lived experience to maximize scientific rigour. This accords with the findings of a review of service user involvement in forensic settings which highlighted the value of positive collaborative relationships.^42^ Threats to external validity can negate the translation of research findings into everyday clinical practice. Such limitations have been described by Kennedy‐Martin and colleagues^51^ who investigated whether RCT participants were representative of “real‐world” patients. Their results indicated that, particularly within the field of mental health research, RCT eligibility criteria could exclude up to 50% of “real‐world” patients. Most often excluded were people with the most severe or complex problems and those considered to be high risk for aggression or suicide. The authors also found evidence suggesting that challenges to successful recruitment of a representative sample could arise from patient factors.^52^ Hence, our ex‐offender SURG who functioned as “by‐proxy” suicidal prisoners were able to guide appropriate amendments during implementation of the study to take into account the real‐world contextual issues experienced by inmates. Further details of how SURG members contributed to the study have been described elsewhere.^28^ Consultation and involvement of patient and public stakeholders in the study of complex interventions have been recommended by the Medical Research Council.^53^ Attention to patient acceptability during research to develop new treatments is important to maximizing future uptake of efficacious interventions.^54^ The findings of our study substantially advance hitherto sparse literature in outlining the factors considered important by ex‐offender populations to achieving their commitment and sustained involvement in the research.

Within our study, there were several key findings which spoke to this issue. Perceptions of a positive milieu during meetings influenced SURG members’ willingness to share their past traumatic experiences as suicidal prisoners. Subsequently, our enhanced understanding of the “real‐world” issues relevant to our target population of suicidal prisoner participants enabled us to design and implement appropriate “user‐friendly” research procedures. This proved to be an important determinant for successful implementation of the study throughout the three‐year duration. SURG members’ willingness to share their “insider intelligence” with researchers unfamiliar with the prison environment positively influenced many aspects of study. For example, in the training of research assistants to recruit and interview participants; in reinforcing the need for researchers to exercise vigilance to maintain their personal safety when interviewing potentially dangerous prisoners; by helping to design comprehensible participant information sheets; and by guiding the sequencing of various components of the trial therapy.^28^

Psychological therapies can only be developed and successfully implemented if communication between the therapist and the client is optimized. Our study has illustrated some generic and transferable mechanisms for optimizing communication with ex‐offender populations which can be used by all professionals including those from healthcare, prison and criminal justice settings. These include the need to form a “connection” with *word deleted* forensic individuals by establishing shared goals bonded by mutual respect, valuing the individual's perspective by listening, tolerance of views and lifestyles that may be very different to one's own.

Armstrong and Ludlow^55^ elucidate the similarities of the overarching aims of universities and statutory offender management schemes as both seek to invest in people, support inclusivity and aim to enrich communities by social transformation. However as the authors point out, “*social growth is achieved through individual growth*”^56^ (p9). It is, therefore, not insignificant that our ex‐offender SURG members revealed that an unintended outcome of their involvement with PROSPeR involved a move away from crime.

Finally, a further consideration is the personal benefits to ex‐offender service users of involvement in studies such as PROSPeR. Ex‐offenders are likely to experience challenges such as reintegration into conventional society, financial hardship and restrictions on employment opportunities due to being imprisoned. Our work showed that our SURG members perceived a range of benefits from being part of this group which nurtured their enthusiasm and desire for remaining with, and actively contributing to the project.

### 4.1 Strengths

This study has contributed to the knowledge base in the underresearched area of service user involvement in prison mental health. Our study offers valuable insights for other researchers of effective ways of involving ex‐offender service users and other “hard‐to‐reach” populations. Our findings suggest that good practice in service user involvement may extend impact beyond the intended outputs of improving the quality of the research to also impacting on the lives of service users that can lead to transformational personal change.

Considerable attention to ensuring scientific rigour was invested in the current study: (i) interviews were conducted by a researcher who was external from the PROSPeR study thereby minimizing potential social desirability bias; (ii) to ensure high‐quality interview data, and with reference to Yardley's principles,^51^ supervision of the researcher addressed issues of contextual sensitivity,^51^ ethical research^57^ and reflexivity^58^; and (iii) researcher triangulation involving several levels of independent and multiple coding processes preceded final agreement of themes.^56^

### 4.2 Limitations

Although our sample comprised only four participants, this represented the entire membership of the SURG, who between them had over 50 years experience of imprisonment. A prevailing feature of IPA is a focus on ideography with small homogenous samples.^29^ Although our sample was small, the level of rich detail of the experiences of this unique population of ex‐offenders is striking. In keeping with the aims of qualitative research, we do not make any claims for empirical generalizability^29^ of our findings. We do however suggest the potential for “theoretical transferability” of an appropriate level of our findings based on assimilation of readers’ own professional knowledge and experience.^29^, ^59^

The organizational constraints pertaining to the PROSPeR study were immense, and we believe the SURG members were highly influential to its success. There are challenges to recruiting ex‐offender service users who are willing to get involved in research and we believe this may be the first and possibly only such group currently in existence. As such our sample size reflects, the very small pool of ex‐offenders contributing to research currently. Further studies with ex‐offender service user consultants are required to elicit a wider range of experiences.

## 5 CONCLUSIONS

This study has shown that it is possible to engage and work effectively with ex‐offender service user consultants and outlines important indicators of the conditions under which relationships flourish, benefiting both the research process and the service user consultants themselves. Our findings indicate that other prison researchers should be optimistic about successfully involving ex‐offender service users in research projects.

## CONFLICT OF INTEREST

The authors declare that there is no conflict of interest.
